# High Preoperative Plasma Fibrinogen Independently Predicts a Poor Prognosis in Patients with Nonmetastatic RCC

**DOI:** 10.7150/jca.40961

**Published:** 2020-02-10

**Authors:** Zhan Wang, Hua Fan, Wenda Wang, Guoyang Zheng, Yu Xiao, Hao Guo, Yushi Zhang

**Affiliations:** 1 Department of Urology, Peking Union Medical College Hospital, Chinese Academy of Medical Sciences and Peking Union Medical College, Beijing, 100730, China.; 2 Department of Pathology, Peking Union Medical College Hospital, Chinese Academy of Medical Science and Peking Union Medical College, Beijing, 100730, China.

**Keywords:** nonmetastatic RCC, preoperative plasma fibrinogen, prognosis, survaval analysis

## Abstract

**Background**: This study aims to determine the relationship between preoperative plasma fibrinogen levels and the prognosis of patients with nonmetastatic renal cell carcinoma (RCC), including overall survival (OS), cancer-specific survival (CSS) and progression-free survival (PFS).

**Methods**: We retrospectively analysed the clinical data and prognostic information of 1194 nonmetastatic RCC patients who received radical nephrectomy or nephron-sparing surgery between 2005 and 2015 at our institution. Serum was collected for fibrinogen detection in the week prior to curative operation, and prognostic information was regularly collected by specially trained personnel. The cut-off value of the preoperative plasma fibrinogen level was defined by receiver operating characteristic (ROC) analysis. The chi-square test was used to analyse the association between preoperative fibrinogen level and clinical characteristics. Kaplan-Meier analysis was used to calculate survival curves, and significant differences were determined by the log-rank test. Other significant prognostic factors were evaluated by the Cox multivariate proportional hazard model.

**Results**: The median follow-up period after radical or partial nephrectomy was 42.4 months (ranging from 0.433 to 146.37 months). The optimal preoperative plasma fibrinogen concentration was 3.975 g/L. The preoperative fibrinogen level was significantly associated with age, pathological T stage, sarcomatoid differentiation, necrosis and vein tumour thrombus (all p<0.05). High plasma fibrinogen levels were related to poor prognosis in terms of OS (p<0.001), CSS (p<0.001) and PFS (p<0.001). Multivariate analysis showed that the preoperative fibrinogen level remained an independent prognostic factor for OS (HR: 3.22, 95%CI: 1.87-5.55, p<0.001), CSS (HR: 4.12, 95%: 2.15-7.89, p<0.001) and PFS (HR: 3.137, 95%CI: 2.17-4.53, p<0.001).

**Conclusions**: High preoperative plasma fibrinogen level is an independent negative prognostic factor for OS, CSS and PFS in patients with non-metastatic RCC. Preoperative plasma fibrinogen could be an ideal indicator for evaluating the outcomes of postoperative patients with nonmetastatic RCC.

## Background

Globally, cancer is the second leading cause of death, as its mortality rate is only lower than that of heart disease 1. Renal carcinoma, one of the most common malignant cancers of the urinary tract, accounts for nearly 5% of all cancers in males and 3% in females, and its incidence has risen steadily over the past 10 years 1, 2. Approximately 90% of renal carcinomas are renal cell carcinomas (RCCs), 80% of which are clear cell tumours 2. Since many patients do not show any specific manifestations, approximately 15%-20% of RCC patients already have been in advanced stage at the first medical consultation, causing a dismal prognosis. Although radical or partial nephrectomy is the standard surgical care for treating patients without metastasis, the prognosis of RCC patients remains poor, especially for patients with regional or distant advanced stage disease 1, 3. Therefore, finding a good prognostic marker is of great significance because we can individually assess risk and adjust our therapeutic strategies. In recent years, a growing body of evidence has shown that preoperative coagulative parameters, especially the plasma fibrinogen level, can perfectly predict the prognosis of RCC 4, 5.

The coagulation pathway may play an important role in cancer pathogenesis since the abnormal activation of coagulation has been observed in many studies. Plasma fibrinogen, one of the most susceptible acute phase reactants created by the liver, can reflect the coagulative state of the whole body. A high preoperative plasma fibrinogen level has been reported to be associated with the poor prognosis of large quantities of malignant tumours, such as lung cancer and gastrointestinal cancer 6, 7. However, data regarding the prognostic significance of fibrinogen levels in renal cell carcinoma are relatively limited, and the mechanism remains unknown.

Hence, the goal of our research was to investigate the prognostic value of preoperative plasma fibrinogen in nonmetastatic RCC patients. Additionally, we aimed to determine the relationship between preoperative fibrinogen levels and clinicopathological characteristics.

## Methods

### Patients and clinicopathological data

We retrospectively collected the clinical and pathological data from 1497 consecutive patients between 2005 and 2015 at our institution. The study was approved by our institutional ethical review board, and all the participants enrolled had already provided informed consent. The seventh edition of the tumour-node-metastasis (TNM) criteria was used for tumour staging, and other clinical information, such as sex and age, was gathered from medical records.

The criteria for inclusion in this study are mentioned below: 1) patients received partial or radical nephrectomy and were diagnosed with renal cell carcinoma by more than two professional pathologists; 2) all the preoperative routine examinations found no distant metastasis; 3) the plasma fibrinogen level was measured by our blood test laboratory within a week before the operation; 4) the follow-up information was regularly updated and complete; 5) patients did not receive any other kind of therapy before the operation; 6) patients did not have other malignant tumours or coagulation disorders before operation. Finally, we enrolled 1194 patients in our study (171 patients were lost to follow-up; 32 patients had metastasis before surgery; 54 patients did not have their preoperative fibrinogen measured; 11 patients received preoperative targeted therapy; 35 patients in total had other malignant diseases or severe coagulative disorders).

All patients received computed tomography (CT) of the chest, abdomen and pelvis before the operation to exclude distant metastasis. PET-CT was performed if the patients show any distant metastatic signs, such as ostealgia and haemoptysis. Follow-up abdominal CT and laboratory examinations were carried out at our hospital every 3 months, starting from the operation date, during the first 2 years, every 6 months during the third and fourth postoperative years, and every 12 months thereafter.

Follow-up data indicated that 165 patients progressed. Among them, 33 patients received a second operation only. Another 39 patients were treated with molecular targeted therapy only, 2 with immunotherapy only, 10 with radiotherapy only and 4 with chemotherapy only. The other patients whose disease progressed received multiple therapeutic strategies.

We collected the survival information from the electronic patient records of our institution or from the electronic records of any other accessible hospital in our city. Missing data were retrieved by telephone interviews with patients or their family members. Overall survival (OS) was defined as the time (in months) between the date of operation and the date of patient death from any cause. Death was be divided into two kinds: cancer-related or not cancer-related. All deaths of patients with confirmed metastatic RCC at any time were considered to be cancer-related. Cancer-specific survival (CSS) was defined as the time (in months) from the date of surgery to the date of cancer-related death. Progression-free survival (PFS) was defined as the time (in months) from the date of surgery to the date of death or progression (recurrence or metastasis) of RCC confirmed by radiology or histology.

### Statistical analysis

Receiver operating curve analysis was used to find the optimal threshold value of fibrinogen. A chi-square test was used to evaluate the relationship between preoperative plasma fibrinogen and clinicopathological characteristics. Univariate analysis (Kaplan-Meier curve) was used to find possible prognostic predictors. In addition, to certify the parameters that were significant in predicting patient prognosis, a multivariate analysis was performed according to the Cox proportional hazard regression model. The statistical analyses were performed by using SPSS version 23.0 and GraphPad Prism 5 for Windows. The p values were 2-sided, and a p <0.05 was considered statistically significant.

## Results

### Patient baseline characteristics

Finally, 1194 patients were included in the analysis, 67.4% (805/1194) of whom were male, and the mean age was 53.74 years (range 15 to 86). Among all the patients, 51.6% (617/1194) received laparotomy, and 55.2% (659/1194) received radical nephrectomy. In addition, the median duration of follow-up was 42.4 months (ranging from 0.433 to 146.37 months), and the mean preoperative plasma fibrinogen level was 5.31±27.25 g/L (range 1.16-471 g/L). The baseline characteristics are shown in Table 1.

### The optimal threshold value of fibrinogen

**Figure 1:**
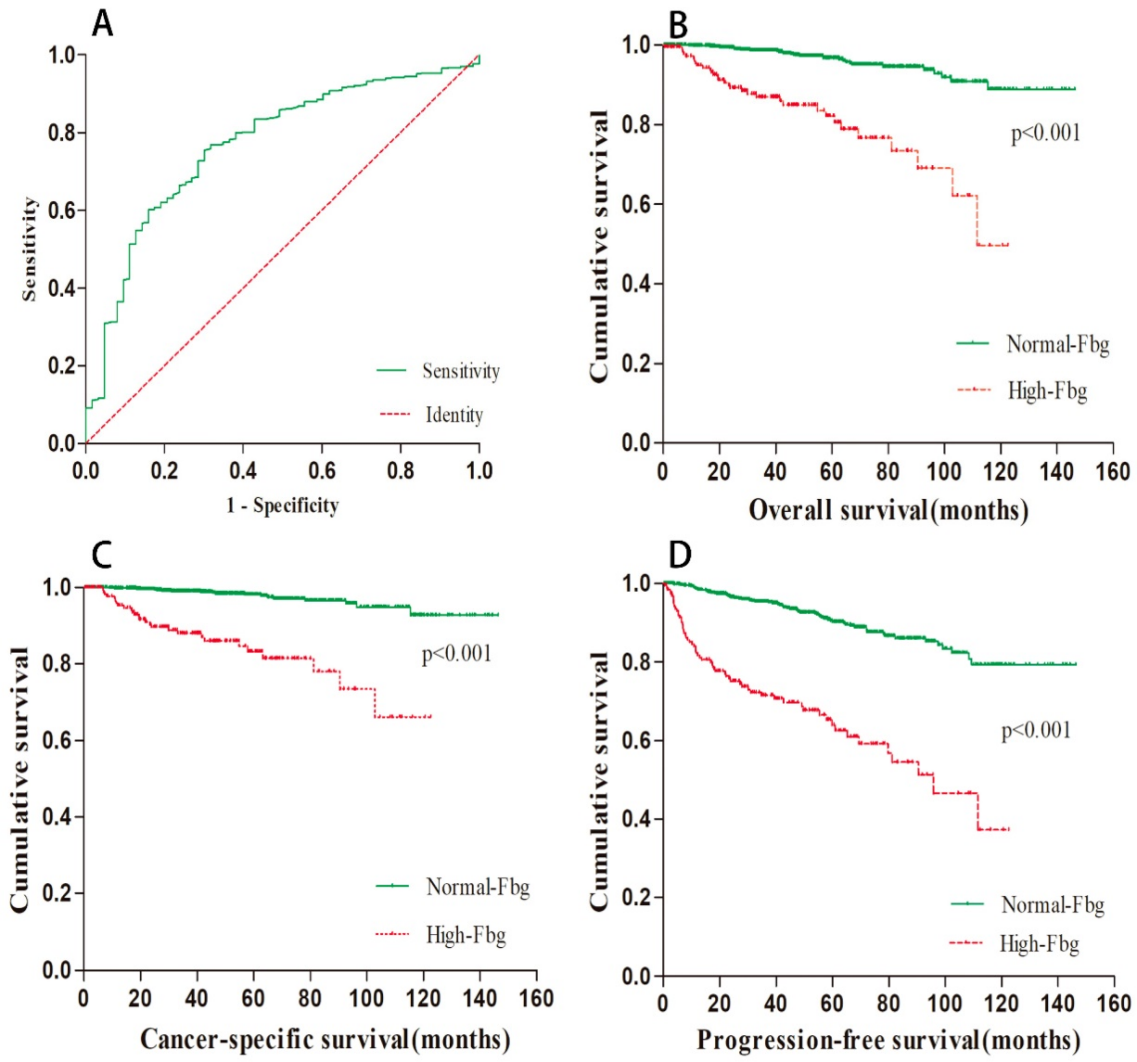
Relationship between preoperative Fbg and survival data. (A).ROC curve of Fbg. Survival curve regarding OS(B), CSS(C) and PFS(D) according to Fbg level.

The mean preoperative plasma fibrinogen level was 5.31±27.25 g/L (range 1.16-471 g/L), and the area under the ROC curve was 0.769. Using ROC analysis, the optimal cut-off value for fibrinogen concentration was 3.975 g/L. Among the 1194 patients, 192 (16.08%) patients had elevated preoperative fibrinogen levels. The ROC curve is depicted in Figure 1A.

### The relationship between preoperative fibrinogen level and the clinical characteristics

In our study, we also investigated the relationship between the preoperative fibrinogen level and the clinical characteristics. Based on the ROC curve, we divided the patients into two groups (the high and normal preoperative groups). The final analytical results are shown in Table 2. From the table, we could clearly conclude that high preoperative fibrinogen level is significantly associated with older age, higher T stage, sarcomatoid differentiation, necrosis and vein tumour thrombus. However, there was no significant association between preoperative fibrinogen level and sex, pathological tumour type, hypertension, diabetes mellitus or smoking.

### Survival analyses based on preoperative fibrinogen level

The median follow-up period was 42.4 months. At the end of our follow-up, 63 (5.28%) patients died, and 47 (3.94%) of them were due to cancer-specific causes. Based on the cut-off value of preoperative fibrinogen concentration, we divided all patients into two groups: the high group and the normal group. Kaplan-Meier survival curves were applied to compare the survival of the two subgroups in terms of overall survival, cancer-specific survival and progression-free survival. The results are presented in Figure 1B-D. As shown in the picture, a higher preoperative fibrinogen level significantly predicted a worse OS (p < 0.001), CSS (p < 0.001) and PFS (p < 0.001).

### Univariate and multivariate Cox regression analyses regarding the significant parameters

To identify the significant factors that may affect the prognosis of RCC patients without distant metastasis, univariate analysis was applied, and the results are shown in Table 1. From the table, we found that age, surgery method, surgery type, pathological T stage, pathological tumour type, sarcomatoid differentiation, tumour necrosis, vein tumour thrombus and preoperative fibrinogen level were all associated with OS, CSS and PFS. However, sex, hypertension, diabetes mellitus and smoking did not have a significant influence on the prognosis of the enrolled RCC patients. We then included all the significant factors into multivariate Cox regression analyses and discovered that the preoperative fibrinogen level remained an independent prognostic predictive factor for OS, CSS and PFS. The results are presented in Table 3.

## Discussion

In our study, we retrospectively analysed the clinical data of 1194 consecutive RCC patients without distant metastasis who received partial or radical nephrectomy at our institution. Based on the receiver operating characteristic curve between preoperative fibrinogen and overall survival, we used 3.975 g/L as the optimal cut-off concentration. The univariate and multivariate analyses revealed that 1) the high preoperative fibrinogen level was significantly associated with older age, higher pathological T stage, sarcomatoid differentiation, tumour necrosis and vein tumour thrombus and that 2) age, surgery method, surgery type, pathological T stage, pathological tumour type, sarcomatoid differentiation, tumour necrosis, vein tumour thrombus and preoperative fibrinogen level were associated with OS, CSS and PFS. However, a high preoperative fibrinogen level remained an independent predictor of poor prognosis for nonmetastatic RCC patients, regardless of OS, CSS and PFS.

Numerous previous studies have discovered that the coagulation system is overly activated in many cancers, such as lung cancer 8, 9, hepatocellular carcinoma 10, 11, pancreatic cancer 12, and oesophageal cancer 13, 14 and so on, but data about cancers of the urinary system, especially renal cell carcinoma, are still lacking. Jun Du 15 enrolled 286 patients receiving radical nephrectomy and retrospectively evaluated the association of preoperative plasma fibrinogen level with the clinicopathological parameters and survival. As a result, they discovered that high plasma fibrinogen was positively related to Fuhrman grade, tumour size, and T stage, and that it was an independent prognostic factor for disease-free survival and OS. However, due to their relatively small sample size, their conclusion may not be persuasive. Later, M Pichler, etc 16 included 994 consecutive non-metastatic RCC patients from a tertiary academic centre and drew a conclusion similar to that of Jun Du. Takeshi Sasaki 5 found that pretherapeutic plasma fibrinogen levels in the metastatic group were significantly higher than those in the non-metastatic group (p < 0.001), and multivariate analysis revealed that fibrinogen (p = 0.036) was an independent prognostic factor for survival in nonmetastatic RCC patients. Later, several studies sporadically identified similar results 17, 18. To our knowledge, our study is the largest and most comprehensive study of Asian patients focusing on the relationship between preoperative fibrinogen and the clinical characteristics and survival of non-metastatic RCC patients. In addition, to the best of our knowledge, our study is the first to find that the preoperative plasma fibrinogen level is also associated with vein tumour thrombus, which tends to predict a worse survival. According to the results of our analyses, it is sensible to stratify RCC patients better and carry out different therapeutic strategies to improve their clinical prognosis.

Although the relationship between the preoperative plasma fibrinogen concentration and the prognosis of cancers has been identified by many studies, the underlying mechanism is still unclear. However, the role of fibrinogen in tumour progression has been identified by many studies. Researchers found that the sustained adherence of tumour cells in the lungs was weakened if fibrinogen was deficient and that thrombin may enhance the metastatic potential of circulating tumour cells possibly through a fibrinogen-independent mechanism 19. Further experiments revealed that spontaneous metastasis relied on the active function of fibrinogen, as fibrinogen could facilitate the stable adhesion or survival of metastatic emboli after tumour cell intravasation 20. In 2000, Abha Sahni explained that through binding up to soluble fibrinogen, VEGF could promote fibrinogen's capacity to support endothelial cell proliferation, which was critical to vascular development and the metastasis of tumour cells 21. It has also been demonstrated that the migration of bladder tumour cells may be enhanced by fibrinogen through its interaction with cellular calcium-dependent adhesion molecule 1 22. In addition, A. Sahni's work demonstrated that fibrinogen endogenously synthesized by extrahepatic epithelial cells could promote the growth of lung and prostate cancer cells through interaction with fibroblast growth factor-2 23. Sheng Zheng et al illustrated that fibrinogen had the ability to block the formation of an effector-target conjugate to regulate NK cytotoxicity because it enhanced the adhesion between tumour cells and platelets, thus forming a dense layer around tumour cells 24. Fei Zhang et al demonstrated that fibrinogen could upregulate the expression of p-PTEN, activate the AKT/mTOR signalling pathway and lead to the acquisition of EMT phenotypes in ESCC 25.

However, it is necessary to note that high fibrinogen levels may be caused by systemic inflammation secondary to tumour progression. Fibrinogen is a plasma glycoprotein synthesised by the liver that is affected by systemic inflammation. Recently, numerous studies have revealed that inflammatory markers such as C-reactive protein (CRP), erythrocyte sedimentation rate (ESR), and neutrophil/lymphocyte ratio (NLR) are also significantly associated with tumorigenesis and affect the survival of a great number of cancers 26-28. Therefore, as one of the most common acute phase inflammatory markers, the fibrinogen level may just be a superficial sign of inflammation related to cancer.

As a retrospective study, there are many limitations to our research. First, our study is a single-centre and retrospective analysis by nature. Second, due to some restrictions, it is impossible for us to include all possible prognostic factors in the Cox multivariate proportional hazard model. Therefore, we could not completely rule out the influence of some undetected confounding factors, such as LDH and plasma Ca^2+^. Third, although we excluded the patients who received multiple preoperative therapies, we could not eliminate differences in postoperative therapy, which may have affected the ultimate results. In addition, we only investigated the relationship between preoperative fibrinogen and nonmetastatic RCC, and our conclusion is not applicable to all RCC patients. Finally, our follow-up was relatively short, and only 63 (5.28%) patients died by the end of our survey. Therefore, a much longer follow-up is necessary in the future.

## Conclusions

High preoperative plasma fibrinogen level is an independent, negative prognostic factor of OS, CSS and PFS for patients with non-metastatic RCC; this information could help clinical doctors better stratify patients and guide their postoperative therapy. Preoperative plasma fibrinogen could be an ideal indicator for evaluating the outcomes for postoperative patients with nonmetastatic RCC.

## Figures and Tables

**Table 1:** The clinical data of all patients enrolled and the univariant analyses of the survival

Variants	No. patients(%)	overall survival	cancer-specific survival	progression-free
		p value	p value	p value
Sex		0.231	0.417	0.073
male	805(67.4)			
female	389(32.6)			
Age,yrs		<0.001	<0.001	0.001
≥60	353(29.6)			
<60	841(70.4)			
Surgery way		0.002	<0.001	0.028
laparotomy	617(51.7)			
laparoscope	577(48.3)			
Surgery type		0.001	<0.001	<0.001
radical nephrectomy	659(55.2)			
partial nephrectomy	535(44.8)			
Pathological T stage		<0.001	<0.001	<0.001
T1, T2	1088(91.1)			
T3, T4	106(8.9)			
Pathological tumor type		0.035	0.004	0.542
ccRCC	1037(86.9)			
other types	157(13.1)			
HBP%	431(36.1): 763(63.9)	0.206	0.706	0.317
DM%	141(11.8): 1053(88.2)	0.274	0.703	0.624
Smoke%	322(27.0): 872(73.0)	0.558	0.446	0.541
Sarcomatoid differentiation%	15(1.3%): 1179(98.7)	<0.001	<0.001	<0.001
Tumor necrosis%	143(12.0): 1051(88.0)	<0.001	<0.001	<0.001
Vein tumor thrombus%	42(3.5): 1152(96.5)	<0.001	<0.001	<0.001
Preoperative Fbg level#	192(16.1): 1002(83.9)	<0.001	<0.001	<0.001

1.ccRCC: clear cell renal cell carcinoma;2.The character “%” means: the number of (existence: inexistence );3. The character “#” means: the number of ( high : normal): 4.Fbg : fibrinogen; 5.HBP: high blood pressure; 6.DM: diabetes mellitus.

**Table 2:** Relationship between preoperative Fbg and clinical characteristics of enrolled RCC patients

Variants	Normal preoperative Fbg level n(%)	High preoperative Fbg level n(%)	p value
Sex			0.808
male	677(56.70)	128(10.72)	
female	325(27.22)	64(5.36)	
Age,yrs			0.014
≥60	282(23.62)	71(5.95)	
<60	720(60.30)	121(10.13)	
Pathological T stage			<0.001
T1, T2	942(78.89)	146(12.23)	
T3, T4	60(5.03)	46(3.85)	
Pathological tumor type			0.954
ccRCC	870(72.86)	167(13.99)	
other types	132(11.06)	25(2.09)	
HBP			0.154
existence	353(29.56)	78(6.53)	
inexistence	649(54.36)	114(9.55)	
DM			0.683
existence	120(10.05)	21(1.76)	
inexistence	882(73.87)	171(14.32)	
Smoke			0.567
existence	735(61.56)	137(11.47)	
inexistence	267(22.36)	55(4.61)	
Sarcomatoid differentiation			<0.001
existence	4(0.34)	11(0.92)	
inexistence	998(83.58)	181(15.16)	
Tumor necrosis			<0.001
existence	89(7.45)	54(4.52)	
inexistence	913(76.47)	138(11.56)	
Vein tumor thrombus			<0.001
existence	14(1.173)	28(2.345)	
inexistence	988(82.747)	164(13.735)	

1. ccRCC: clear cell renal cell carcinoma; 2.Fbg : fibrinogen; 3.HBP: high blood pressure; 4.DM: diabetes mellitus.

**Table 3:** The multivariant analyses of the prognostic factors regarding OS, CSS and PFS

Variants	Overall Survival			Cancer-specific Survival			Progression-free Survival		
	HR	95%CI	p value	HR	95%CI	p value	HR	95%CI	p value
Age	3.249	1.927-5.478	<0.001	1.957	1.068-3.587	0.030	1.452	1.031-2.044	0.033
Surgery way	0.427	0.221-0.824	0.011	0.488	0.226-1.053	0.068	0.719	0.489-1.056	0.093
Surgery type	0.643	0.326-1.268	0.643	0.508	0.209-1.235	0.508	0.591	0.382-0.914	0.018
Pathological T stage	1.756	0.942-3.271	0.076	2.069	1.021-4.193	0.044	1.613	1.036-2.512	0.034
Pathological tumor type	1.728	0.903-3.304	0.099	2.328	1.147-4.723	0.019	1.109	0.683-1.802	0.676
Sarcomatoid differentiation	3.550	1.392-9.054	0.008	4.599	1.747-12.106	0.002	2.562	1.177-5.576	0.018
Tumor necrosis	1.800	1.021-3.174	0.042	1.812	0.946-3.473	0.073	1.851	1.256-2.726	0.002
Vein tumor thrombus	3.170	1.588-6.327	0.001	3.544	1.672-7.514	0.001	1.904	1.104-3.282	0.021
Preoperative Fbg level	3.221	1.870-5.546	<0.001	4.116	2.147-7.889	<0.001	3.137	2.172-4.533	<0.001

1.OS: overall survival; 2.CSS: cancer-specific survival; 3.PFS: progression-free survival; 4.HR: hazard ratio; 5. CI: confidence interval.

## Abbreviations

ccRCC: clear cell renal cell carcinoma
CI: confidence interval
CSS: cancer-specific survival
DM: diabetes mellitus
Fbg: fibrinogen
HBP: high blood pressure
HR: hazard ratio
OS: overall survival
PFS: progression-free survival
